# A major hurdle in the elimination of urogenital schistosomiasis revealed: Identifying key gaps in knowledge and understanding of female genital schistosomiasis within communities and local health workers

**DOI:** 10.1371/journal.pntd.0007207

**Published:** 2019-03-21

**Authors:** Vida Ami Kukula, Eleanor E. MacPherson, Irene Honam Tsey, J. Russell Stothard, Sally Theobald, Margaret Gyapong

**Affiliations:** 1 Dodowa Health Research Centre, Ghana Health Service, Dodowa, Ghana; 2 Malawi-Liverpool-Wellcome Trust, Blantyre, Malawi; 3 Department of International Public Health, COUNTDOWN Consortium, Liverpool School of Tropical Medicine, Liverpool, United Kingdom; 4 Department of Parasitology, Liverpool School of Tropical Medicine, Liverpool, United Kingdom; 5 COUNTDOWN Consortium, Institute for Health Research, University of Health and Allied Sciences, Ho, Ghana; George Washington University, UNITED STATES

## Abstract

**Background:**

Urogenital schistosomiasis is endemic throughout Ghana with elevated infection levels in certain areas e.g. Lake Volta Region. While the primary focus of the national control program is on mass drug administration of praziquantel to school-aged children, Female Genital Schistosomiasis (FGS), a disease-specific affliction of girls and women, has been largely overlooked. To better focus future actions, our study investigated the perceptions, knowledge and understanding of FGS amongst community members and health providers.

**Method/Principal findings:**

We used qualitative methods including 12 focus group discussions and 34 in-depth interviews. We purposively selected 16 communities along the Lake Volta in the Shai-Osudoku District. Participant selection was based on gender, age and occupation; providing an opportunity to explore community understanding of FGS through participants own words and perceptions. Awareness of schistosomiasis was reported and is commonly experienced among children (12–17 years) and younger adults (18–25 years) in the study communities but is typically considered a boy’s disease. Knowledge of FGS was lacking in women, girls and front-line health workers. There was a general misconception that FGS may be the result of sexual promiscuity. Adolescent girls reporting vaginal discharge and itching were often stigmatized by health workers and treated for sexually transmitted infections. Limited alternatives to the river as key source of water meant that all members of the community faced the regular risk of schistosomiasis.

**Conclusion/Significance:**

There is a clear imperative for the national control program to better engage on FGS and implement interventions to meet girls and women’s needs. The key consideration is to integrate more adequately preventive services with sexual and reproductive primary health care with future training of health workers for improved management of FGS cases. More broadly, harmonizing the portfolio of all actions on FGS is needed, especially with a call for improved access to safe water and sanitation for all those at current or future risk.

## Author summary

Female Genital Schistosomiasis (FGS) is a serious, chronic gynecological condition that affects up to 56 million African women and girls. To date, FGS has been mostly overlooked within national neglected tropical disease control programs and associated health systems in sub-Saharan Africa. Furthermore, knowledge and understanding of FGS within affected communities themselves are often incomplete and confused with other ailments. Using qualitative research methods, we explored the knowledge and understanding of girls and women on FGS, as well as, local health care providers’ knowledge and practice relating to FGS. While, our work found that FGS was rarely on the radar of suspicion for health workers, more importantly, adolescent girls were often stigmatized by health care workers when they sought care in public health facilities owing to misdiagnosis of the disease as sexually transmitted infections. Our work speaks to the urgent need for national control programs to expand their remit outside current focus of annual provision of praziquantel to in-school children and integrate such treatments within sexual and reproductive health services available in primary health care services. Ultimately, urogenital and intestinal schistosomiasis is a disease of poverty and there is an urgent need to extend access to improved water and sanitation facilities to all affected communities.

## Introduction

Schistosomiasis is an important parasitic disease that affects the health and well-being of over 200 million people throughout the world [1], causing some 3.3 million disability-adjusted life-years [2]. Over 95% of the global burden of schistosomiasis is found in sub-Saharan Africa where two forms of schistosomiasis exist, intestinal and urogenital [3], More than half of those infected, at least on the basis of schistosome eggs in the excreta, are school-aged children [4].

Urogenital schistosomiasis is caused by infection with Schistosoma haematobium, a dioecious blood fluke that utilizes specific freshwater snails of the genus Bulinus as obligate intermediate hosts. Adult female flukes lay terminal-spined eggs, often in copious amounts each day that become partially lodged or later trapped within all organs of the urogenital tract. The eggs that perforate and cross the bladder wall, co-transit with leakage of venous blood. This leads to the cardinal sign of urogenital schistosomiasis known as macro-hematuria or red urine. Through poor environmental sanitation control, eggs that reach freshwater subsequently hatch to release a miracidium, that actively searches and penetrates a freshwater snail where it undergoes asexual reproduction. After sufficient development within the infected snail, to complete its lifecycle, a different larval stage emerges, the cercaria, often produced in enumerable amounts each day, and is able to cross unbroken skin directly. Water contact activities such as washing and bathing in water where cercariae are present enables the schistosome to gain entry into the human host. Indeed, any demographical groups, irrespective of age or gender with unsafe water contact is at risk of infection [5].

Like all neglected tropical diseases, schistosomiasis is a disease of poverty. The broader social environment in which people live and work is key to understanding vulnerability to the disease [6, 7]. Those at greatest risk are typically in rural environments where daily access adequate sanitation and hygiene is poor [2, 7]. For many, accessing and using water is a vital part of both household survival and many livelihood activities in rural communities [8, 9]. This way of life means that most people living in communities where schistosomiasis is present face daily risk of infection [1]. There are, however, key gendered roles and norms that place different groups of men, women, girls and boys at differential risk. The gendered roles of women and girls within the household including chores such as washing clothes and dishes, collecting water for household consumption, puts them at risk from near continual water contact in fulfilling these roles [9, 10]. In many communities, it is often socially acceptable for adolescent boys to play and swim in rivers and lakes, thus increasing their risk [10]. Livelihood activities such as fishing and agriculture often require contact with infested bodies of water. Understanding gendered risks different groups face is important for developing strategies to prevent transmission, as indeed anyone who is shedding eggs can be responsible for infecting snails.

### Global strategy for controlling schistosomiasis

The WHO recommended strategy for controlling schistosomiasis focuses on the large-scale periodic administration of praziquantel to entire populations (targeting school-age children, adults at risk including pregnant women, lactating women and people whose occupation put them in contact with infested water);

While the WHO strategy recommends reaching both school-based and community-based programs, a priority of the global program has been on the mass drug administration of praziquantel to school-age children [11]. Gray and colleagues [2010] highlight that praziquantel based control programs have only a temporary effect on transmission and are limited in their potential to interrupt disease transmission in the long-term [12]. Bruun and Aagaard-Hensen [2008] argue that the narrow focus on distributing drugs to in-school children also means that out-of-school children are overlooked. This approach also fails to address the broader social environment that drives transmission and does not strengthen capacity within the health system to treat and manage schistosomiasis. Indeed, control of schistosomiasis is seen by many through a narrow lens of preventive chemotherapy rather than from the vista of the patient with advanced life-threatening disease e.g. kidney failure or hematemesis. This creates an unfortunate polarization and underlying health systems tension in deciding which interventions are best applied when, where and why.

### Female genital schistosomiasis (FGS)

Female Genital Schistosomiasis (FGS) is a specific disease entity of urogenital schistosomiasis. FGS occurs when the damage of schistosome eggs specifically occurs in any part of the female reproductive system. The damage can range from the formation of sandy patches in the uterus and cervix to the development of vulva nodules in the lower genital tract [13, 14]. The disease causes an array of gynecological symptoms in women and adolescent girls which include vaginal discharge, vaginal bleeding, vaginal discomfort and pain during sex [15]. Girls as young as ten have reported symptoms of FGS-associated vaginal discharge [16]. When untreated, the damage that FGS causes include infertility, miscarriage, ectopic pregnancies, spontaneous abortions, prematurity, social stigma, depression, vulva nodules, genital and cervical lesions [15]. There is evidence that FGS can increase vulnerability to HIV and Human Papillomavirus (and the corresponding risk of cervical cancer) [17, 18]. A significant gap in accurate epidemiological data on the disease exists with estimates ranging from between 20 and 150 million girls and women being at-risk [14, 15]. Despite causing significant suffering to millions of women and girls, FGS has been overlooked [15]. In many endemic areas, there is inadequate detection, treatment and prevention services for women and girls living in affected areas. Christinet and colleagues [2016] note that there is a lack of capacity in the health care system to effectively diagnose and manage patients [15]. In addition, there is a paucity of academic work exploring health workers understanding and management of the disease. Ghana has a well-established neglected tropical disease control program which distributes praziquantel (through Mass Drug Administration) for the control of Schistosomiasis in school children.[19]. Despite these efforts, Schistosomiasis can be found in all ten regions of the Country, with the disease mostly found in rural and some urban communities [20]. This research project was nested in a larger study called the COUNTDOWN (Calling Time on Neglected Tropical Diseases) project which is a multidisciplinary, multi-country study exploring strategies to improve the control of neglected tropical diseases. This study aims at understanding community and health provider perceptions and practices relating to FGS in rural communities in Southern Ghana with a high prevalence of Schistosomiasis. It contextualises the findings in the broader socio-economic, gender, political and economic structures that shape behaviour.

## Methods

### Ethical statement

Ethical approval for the study was obtained from the Ethics Review Board of Ghana Health Service, the Institutional Review Board of Dodowa Health Research Centre and the Research Ethics Committee of the Liverpool School of Tropical Medicine. Written and verbal consent was given by participants. Participants, less than 18 years gave assent while their parents and guardian gave consent for them to participate.

### Study setting

**Fig 1:**
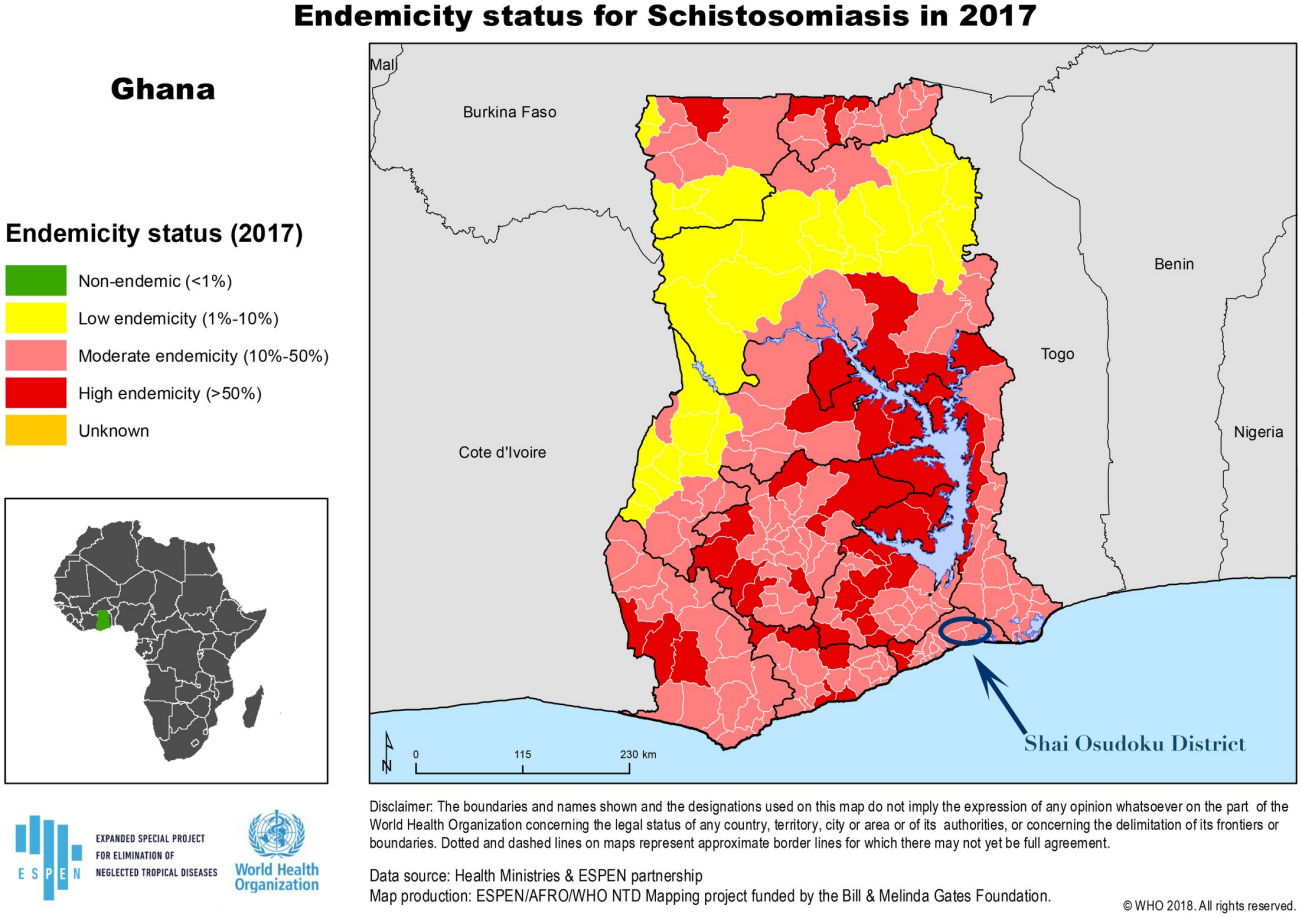
Endemicity status for schistosomiasis in Ghana [20].

The study was conducted in Osudoku in the Greater Accra region of Ghana. Osudoku is a sub-District of Shai-Osudoku (see Fig 1). We purposively selected fifteen communities, which had a high prevalence of 54.8% and 60.0% urogenital schistosomiasis [21]. While, we did not have exact figures for FGS, based on other studies in the region, this is likely to be approximately 11% [22, 23]. The predominant livelihoods of people living in the communities are farming and fishing with the Volta river as the main source of water for both household and economic activities.

In the selected communities, in-line with the global strategy to control schistosomiasis, the mass administration of praziquantel was being delivered to in-school children [11]. Access to praziquantel for other groups such as preschool-aged children and women has been poor, with drugs rarely distributed beyond in school programs.

### Study design

The central aim of this study was to understand the beliefs and practices of community members and health care workers in relation to FGS We used qualitative research methods because they provide research participants with the opportunity to express their own lived realities and allow researchers to understand how social experience is created and understood in everyday life [22, 23]. We conducted the study between June and August 2017 and used semi-structured interviews and focus group discussions for data collection. During the focus group discussions with younger participants, we used vignettes. A vignette is a participatory tool that uses hypothetical scenarios of a person which unfold through a series of stages to stimulate discussion of the topic under study [24]. We developed a vignette representing realistic experiences of a schistosomiasis-infected girl, drawing her daily activities and enabling discussions around; knowledge on schistosomiasis; health risk of schistosomiasis; symptoms of schistosomiasis in girls and health-seeking behavior for schistosomiasis.

Development of our sample frame was guided by the principle of maximum variation to ensure we included a wide range of perspectives [25]. We purposively sampled participants based on age and gender. Children (12–17 years) and adults (above 17 years) living and working in the study communities were selected. We included health care providers (medical doctor, midwives, general nurses, community health officers, community disease control officers), school teachers, traditional chiefs, traditional birth attendants (TBAs), traditional queen mothers, community opinion leaders, community medicine sellers, adults and adolescent girls. We also sampled men and women involved in livelihood strategies that specifically required regular contact with water. We undertook a total of 34 semi-structured interviews and 18 FGDs (6 which included vignettes). Four trained research officers including the first and third authors conducted the interviews in the main local languages (Ewe, Ga and Dangme).

### Data analysis

We used the framework approach to qualitative research analysis [26]. At the end of each day of data collection, the research team held debriefing sessions. During these daily sessions, the team reviewed the interview notes and listened to the audio recordings to reflect on the key themes that emerged from the interviews. This strategy allowed new emerging themes to be identified and incorporated into the interview guides for subsequent interviews that ensured rich textured data quality and continued feedback. All audio recordings were transcribed verbatim and translated into English. The transcripts were imported into the qualitative software NVIVO version 11 and the program was used to aid data analysis by coding against the framework. The team then developed an initial thematic framework which was used to code the transcripts and all materials including field and debriefing notes. The framework was then updated as new themes emerged.

## Results

The results are presented around the five key interlinked themes that emerged in our analysis: community and health workers’ beliefs about schistosomiasis, gendered understandings of symptoms for schistosomiasis, treatment and care-seeking practices for schistosomiasis, knowledge and understanding of female genital schistosomiasis and gender, water usage and risk of schistosomiasis.

### Community and Health-Worker beliefs about schistosomiasis

We found that there was wide recognition that schistosomiasis was present in the communities and that the disease was a concern for health and well-being, particularly for boys and men. Both adult men and women in the communities reported that information about the transmission of schistosomiasis was given by community health workers, NGOs working in the district and media channels including television and radio. As one 56-year fish seller reported, ‘‘I heard about ‘Muozim’ [local name for schistosomiasis] from the community nurse during the last festival at the community gathering where she said we should not be urinating and defecating in the water because it can cause the disease” Adult woman FGD. During the focus group discussions with the younger girls and boys, both school and the local clinic were discussed as key sources for information about the transmission of schistosomiasis.

Across the dataset, all groups sampled identified the Volta Lake as a key source of schistosomiasis. Adult males, females and school pupils further reflected that worms and snails within the water were responsible for the transmission. However, there was a complex understanding of the ways in which schistosomiasis was transmitted and the reasons people became infected which often went beyond biomedical explanations of transmission pathways. An example of this is provided in the quote from a focus group discussion with adult women living and working in the communities:

“When you eat sugar cane from the river bank you will swallow the snail and it will hatch in your stomach and cause schistosomiasis.” Adult Female; focus group discussion.

There was also concern from both male and female participants that schistosomiasis could be transmitted through sexual relationships. In contrast, most health workers’ understandings of the pathway to transmission-related directly to biomedical explanations as outlined in this quote from a health worker:

“Bathing, swimming, washing and drinking contaminated water lead to the disease entering the body leading to schistosomiasis” IDI with Health worker

Some health care providers highlighted the challenges associated with a limited understanding of the transmission dynamics of schistosomiasis by community members. One community drug seller stated that “the community is not well informed about schistosomiasis; most people just know the fact that it is caused by snails in the river we use, as for how you protect yourself and the medicine for treating it, they do not know”. IDI with community drug seller.

### Gendered understanding of symptoms for schistosomiasis

Muozim is the word used in Dangme to mean schistosomiasis. The term literally translates as blood in the urine and there was wide recognition from all groups involved in the study that schistosomiasis caused bloody and painful urination. However, there was a strong sense by a wide number of groups including older men, women, adolescent boys and some community-based health workers that schistosomiasis is an illness that predominately affects men rather than women. This illustrative quote from a Traditional Birth Attendant (TBA) represents some of the discussion on this “there is no such disease for women. Only boys have this disease that causes bloody urine. It is only in boys” IDI TBA. She went on to say that women only see blood when they menstruate or after they give birth.

Older men and women reflected on the idea that women and girls tend to experience a different form of schistosomiasis which did not cause bloody or painful urination. The general perception in the focus group discussions with men was that most women and adolescent girls became infected with the disease through sexual contact. This idea was captured in this illustrative quote by an adolescent boy "I have never heard the girls complain about urinating blood, it is only the boys. Girls have some kind of defense against the schistosomiasis from the lake that affects the boys, only the men can pass it to them'' FGD school boy’s group. A male teacher from one of the schools in the community echoed that; “I think the ladies can only get it when the boys give to them otherwise I do not know how they get it if they do get it at all. But since you are asking these questions, it implies that the girls also suffer from the disease” IDI teacher.

There were also gendered differences in health workers’ perceptions of risk and symptoms of schistosomiasis. Some health workers perceived boys and men to be at higher risk than women and girls. This was due to the association with the passing of blood in the urine with boys and men and not women and girls. One midwife noted that ‘‘I have worked here for 12 years and have never seen a girl report with bloody urine. It is always the boys. I do not think girls get it much or girls urinate blood”. Midwife IDI

However, a smaller number of health workers including a doctor, midwife and a medicine seller on probing, reflected on the serious physical manifestations schistosomiasis can cause in women and adolescent girls. The participants emphasized the gynecological symptoms relating to schistosomiasis including vaginal itching and sores; irregular menstruation; infertility; painful sex; bleeding during sex; vaginal and cervical cancer.

### Treatment seeking and care practices for schistosomiasis

When we asked participants about where they sought care for their condition, most men, women and adolescent boys and girls mentioned using home-based remedies and visiting herbalists as the first action. If symptoms persisted or became more severe, boys and some girls discussed seeking care at the community-based and health planning services(CHPS) compounds. When we probed specifically around care seeking for schistosomiasis most groups stated that there was the provision in the community clinics for treatment for schistosomiasis. However, there were some concerns relating to the effectiveness of the treatment for schistosomiasis from the clinic. An opinion leader said, “the white man’s tablets are difficult to swallow and sometimes it gives you bad effects. I don’t use it for my children when they urinate blood. I use the herbs and it works well”. IDI male opinion leader.

There was a marked difference between adolescent boys’ and girls’ treatment seeking pathways when they suspected they had symptoms of schistosomiasis. Adolescent girls were more reluctant to seek care at government clinics and hospitals whereas adolescent boys talked more openly about seeking such care. Girls’ reluctance to seek care was explained by both the cost of treatment and their concern that the drugs were ineffective. One girl said, “as for me, the moment I saw blood in my feces and itching vagina, I told my mother and she gave me some herbal mixture to drink and insert in my vagina and I was okay within 3 days. My friend used the clinic medicine for months and it did not improve” FGD with Vignette adolescent girls group.

The major barrier for girls and women seeking care at formal health clinics was the stigma. A 17-year-old pupil said, "when the girls report to the clinic with vaginal itching, discharge or pain, the nurses always start to advise you to avoid sex or use a condom for protection. Even sometimes they can tell your mother that you are having sex and so you can get HIV; they will talk for a long time and will not tell you that you have schistosomiasis or request you to do any test but they will say you have STI and give you drugs for STI. That is why we the young girls don’t go to the clinic''. FGD Adolescent female. Her view was generally supported by almost all the other adolescents in the group discussion. A 25-year-old woman also reported that “the nurses are very rude to us young women, my friend once went with vaginal discharge and itching and the nurse told her to change her bad ways else she will one day get AIDS if she keeps sleeping around. She did not even educate us about ‘schisto’ or tell her to go to the lab and test”. FGD, Adolescent female.

During one of the interviews, an adolescent girl provided a specific example of when she reported having gynecological symptoms at the clinic but was chastised by the nurse and given inappropriate treatment. "When I reported to the clinic with bloody urine, vaginal itch, discharge and lower abdominal pain, they referred me to the family planning clinic where the nurse asked me the last time I had sex, number of men I slept with and told me the symptoms were STI. She gave me some medicine and told me to abstain from sex. But I have never had sex in my life, I am only 14 years then. The medicine she gave me did not work and I finally went to the drug store where they treated me and said it was schisto'' IDI Adolescent female.

As noted above, some of the health workers did not believe women or girls could contract schistosomiasis. When asked directly about the management of gynecological symptoms in women, one community health worker confirmed this treatment and said, “When young girls or women come to the clinic with vaginal discharge or vaginal itching, painful urination and weakness, we use the STI treatment guidelines to treat them because we think it is STI”. IDI Community Health Worker.

Mass drug administration of praziquantel was discussed by a limited number of adults and adolescent girls and boys as a source of treatment for schistosomiasis. Yet, a number of groups including parents, teachers and adolescents attending school felt that there was inadequate information on the drugs given to children during the school MDA. There was concern that the health service did not inform the community when the drugs were going to be given in the schools. The school pupils also said the teachers and nurses told them the drugs were for treating intestinal worms rather than schistosomiasis leading to confusion and concern. These limited engagements and lack of clarity on purpose were cited by some parents as the reason they prevented their children from taking the medicines. ‘‘If the medicines they give in the school is to treat against the bloody urine, then it is very good but because we the parents are not informed, our children do not take it” IDI Queen Mother.

### Knowledge and understanding of female genital schistosomiasis

During the interviews, focus group discussions and in-depth interviews we probed specifically about knowledge and understanding of FGS. Although the adult and adolescent females discussed gynecological symptoms experienced by them, they were not able to relate their symptoms to schistosomiasis. None of the community members had heard of female genital schistosomiasis as a specific disease. This was also reflected in the interviews with most front-line health workers who expressed a lack of knowledge on the causes, prevention, transmission, symptoms and complications of FGS. One gynecologist and midwife working in the cervical screening expressed a very good understanding of FGS. However, in this role, they had a high level of training in the management of gynecological diseases. This midwife explained that if the disease is left untreated, it could result in serious complications like infertility and cancer of the cervix: ‘‘the way the urogenital schistosomiasis is, if untreated in the female, the organism can travel to affect the womb and cervix causing infertility, miscarriage, ectopic pregnancy or even cervical cancer. I think all girls and women in this area should be checked and those found to have it should be treated on time to prevent these complications” IDI Midwife. During the interview with the gynecologist, he described how schistosomiasis and FGS were of particular concern for women and said that “it commonly manifests as blood in urine, itchy vagina, irregular menstruation and anemia” IDI Medical Doctor. He further explained that girls and women do not usually report blood in urine because they confuse it with their menstruation.

### Gender, water and schistosomiasis risk

As noted above, most of the participants saw that interaction with the river was a risk for contracting schistosomiasis. When we specifically probed around women and girl’s use of the water body, men and women described how women and girls frequently interacted with the river for washing clothes, bathing and fetching water. This quote highlights some of this discussion “girls and women are always in the water every time. Whenever you go to the river, you will see the women washing, bathing or fetching water. They are always there in the water, putting them at risk.” IDI Queen Mother.

Male community members also saw women and girls spending longer periods in the water during the hot season. In these interviews and focus group discussions, there was a concern that women were less able to handle the heat than men. This quote from a 64-year-old man provides an example of how this was discussed “the women cannot stand the heat during the hot season and so they always spend their free time cooling themselves in the river and this puts them more at risk”. FGD adult male. A male drug seller also described menopause as a key driver of contact with water “my wife and daughters are always in the river when the weather gets hot. She has this women’s sickness they call menopause and sweats a lot and so when the weather gets hot she is always in the river and this action can put her and my girls at risk”.

Men and women described gendered differences in the way open water sources were used. Men were primarily using the river for fishing and bathing. Women and girls used the lake for washing clothes, collecting water for the household and bathing. During our time in the communities, we noted that there were alternative water sources such as boreholes and standpipes. When we probed women directly as to why they did not use these sources they described several barriers including broken boreholes and standpipes, and also user fees which made them unaffordable or a complete lack of infrastructure. Teachers, health workers and opinion leaders also saw the absence of adequate toilet facilities in lakeshore communities as a key driver for the community using the lake for toileting.

One adolescent female noted that was almost impossible to prevent men and women from contracting schistosomiasis because their lives and activities revolve around the Volta Lake. ‘We do not see how we can control or prevent our community from getting schistosomiasis, we live every day in the lake where the snails live and affect us. Everything we do daily like fishing, washing, bathing, swimming is all in the water. We will have to drain the whole water before we will be safe because people go into it every day” FGD adolescent female.

During the interviews and focus group discussions, we asked all participants about the ways we could prevent the transmission of schistosomiasis. The four key strategies identified by participants were limiting access to the lake, providing better education on schistosomiasis, enhancing treatment services and improving access to water and sanitation facilities. In the adult male and female FGD discussions group, they called on the community to control girls and women’s from accessing the river. This included a suggestion by a head teacher that “girls especially, school girls should be banned from the river”. IDI Teacher. During an adult male focus group discussion, some participants suggested having local council laws that ban girls and women from entering the river. ‘‘the government through the local council should put down laws and regulations that will prevent our girls from using the river so that they do not get these dangerous diseases”. FGD adult male.

Two health workers, a teacher and a queen mother argued that diagnosis and treatment for schistosomiasis needed to be extended into the communities beyond school-based mass drug administration and should specifically target women and girls. "I think there should be a community-wide screening of girls and when found with the infection, they should be treated immediately” IDI Community health nurse. ‘‘Why do you give the treatment to only school children if the disease is in the water and everyone gets it. Next time make sure everyone gets the treatment” IDI Teacher. To overcome the barrier of cost the queen mother said, “tell the health service to come into the communities, test the urine of every girl and woman including myself, and anyone with the disease should be treated immediately and free of charge”.

The main preventive and control strategies recommended by the male and female adult and adolescent groups and school pupils included intense health education on schistosomiasis and FGS ‘‘People in this community need education on the disease and how it affects our women. The government needs to bring the health people to educate us” Adult Male FGD. The chief of one of the villages also suggested that the teachers should teach the children not to urinate or defecate in the water and treat the river regularly by killing all the snails and worms in it.

Improving access to safe water supply and improved toilet facilities was also discussed by male and female participants during the focus group discussions. This quote clearly summaries these ‘'we need toilet facilities so that people will stop going to the toilet in the river. They also need to give us boreholes and pipe water so that we will not go into the water to get the disease'' Male FGD.

## Discussion

In the study communities, we found very little knowledge or recognition of FGS by health workers and all community members [men, women, adolescent girls and boys]. The exception to this was a doctor and nurse with specialist training in gynecology. There was a much wider recognition of schistosomiasis by both health workers and community members and bloody urine was a symptom strongly associated with the disease. In the interviews and focus group discussions, understanding of the transmission pathways of schistosomiasis went beyond bio-medical explanation and included sexual transmission. Boys and men were viewed as far more likely to be at risk of infection for schistosomiasis than women and adolescent girls. With some health workers stating they had never seen a woman or girl infected with schistosomiasis. Reflecting these community constructions of risk, when younger girls did report to the clinics they were often stigmatized by health care workers. Girls often faced accusations of sexual promiscuity and being referred for STI treatment even before they had become sexually active. This stigmatization was cited by adolescent girls as a key barrier to seeking care at formal health facilities and a preference for home remedies or herbal solutions. In the household, gendered norms mean that women and girls were responsible for collecting water, washing and bathing and led to near constant interactions with the water. Suggested approaches to control schistosomiasis included providing improved water and sanitation facilities as well as enhanced treatment services that extended into the communities. Some health workers and adult men and women noted the limitations of relying on mass drug administration to in-school children. Parents were particularly concerned about the lack of information provided during mass drug administration campaigns.

To date, there have been very few studies exploring the belief and practices of health workers, women and adolescent girls in relation to female genital schistosomiasis [15, 16]. Most academic work published is in the form of clinical case reports or quantitative assessments of the burden of disease [16, 27]. In a study conducted in the Volta basin of Ghana by Yirenya-Tawiah and colleagues [2011] on the prevalence, knowledge, practices and beliefs on urogenital schistosomiasis, a prevalence rate of 10.6% for female genital schistosomiasis was reported. In responses to structured survey questions, participants reported some confusion around disease transmission with some groups reporting the disease was transmitted by sexual contact or punishment by the gods [28, 29] which confirm findings of this study.

The strong association of schistosomiasis with boys has been identified in other academic studies. Person and colleagues [2016], in their study exploring urogenital schistosomiasis on the Zanzibar islands of Tanzania, found that people saw the disease as normally affecting boys [30]. For boys, the disease did not have associations with severe health outcomes or stigma. Person’s [2016] study also discussed the beliefs of children and adults about the way schistosomiasis was transmitted; also identifying explanations which went the biomedical.

The damage that undiagnosed FGS causes can include severe gynecological complications, irreversible gynecological lesions and an increased risk for HIV women [13, 14]. Christinet and colleagues [2016] note that one of the major gaps in addressing FGS is health care professionals lack the capacity to effectively diagnosis and treat women and girls [15]. This supports our finding on how little awareness health workers had about FGS and the highly stigmatizing experiences some of the women and adolescent girls faced when they went to the clinic. We were unable to identify academic studies that reported qualitative findings around health workers’ beliefs and practices around FGS. However, the two participants with in-depth knowledge were highly specialized medical practitioners and stressed the importance of referring women to specialist services. This will only happen if schistosomiasis and FGS are on health workers’ radar of suspicion.

There have been a number of papers published that have raised concerns about school-based MDA [7, 12, 28, 31], the potential negative impact this approach to MDA can have on a fragile health system and concerns about the way community engagement approaches have been implemented [32]. The approach has consistently missed a large population of at-risk out of school-age children and other young girls and women who also need the intervention as the in-school population. In our study, parents reported concerns about the way the MDA campaign had been communicated to them and health workers noted the limitation of only targeting in school children. Our study speaks to the need to expand access to diagnosis and treatment services beyond in-school based MDA.

Schistosomiasis is a disease of poverty [7]. Women, men, girls and boys become infected in rural African communities due to neglect on the part of local, national and global actors. Gendered roles and relationships shape women, men and girls and boy’s interactions with water; although most community members were at risk of infection with Schistosomiasis. Girls and women were required to access water for bathing, drinking and washing dishes but men and boys also needed to use the water for livelihood activities and bathing. In our study, participants spoke of the lack of alternatives to the river and lake which stopped them from changing their behavior. In rural South Africa, Tanser and colleagues [2018] found a high coverage of piped water in a community decreased a child’s risk of urogenital schistosomiasis infection by eight-fold [2]. To break the cycle of schistosomiasis interventions are required that look beyond MDA for in-school children and address the sub-optimal access to safe water and sanitation. Mwai and colleagues [2016] also emphasized this finding in their work in Kenya on the knowledge and practice for the control of schistosomiasis [33].

There were several limitations to our study. Due to the limited clinical surveillance of FGS, we did not have prevalence rates for the communities we conducted the research in. Therefore, we did not know the extent of the problems of FGS. However, we do know from other studies that these communities were likely to be affected by FGS. We had to probe directly around several areas relating to FGS and this may have shaped participant’s responses. We only introduced prompts once participants had responded to initial open-ended questions. Although interest in Male Genital Schistosomiasis (MGS) is now growing, we did not formally consider this aspect in our work but hope to do so in future [34].

Key recommendations from our study findings include the need for an integrated system of health care delivery that includes FGS in the broader symptomatic and clinical management of sexual and reproductive health programs. This will reduce the long-term harm to women and girls who are mostly the marginalized population. There is the need for a change in the curriculum of nursing and other health professional training to reflect schistosomiasis and other NTDs. While training, education and awareness creation will help reduce the incidence of occurrence, the provision of physical structures such as potable drinking water and adequate toilet facilities for the communities are vital to prevent schistosomiasis transmission.
